# INCOME AND WELL-BEING IN OLD AGE—SYSTEMIC AND REGIONAL FACTORS IN A COMPARISON BETWEEN GERMANY AND POLAND

**DOI:** 10.1093/geroni/igad104.0224

**Published:** 2023-12-21

**Authors:** Monika Oczkowska, Ellam Kulati, Michal Myck

**Affiliations:** Centre for Economic Analysis CenEA, Szczecin, Zachodniopomorskie, Poland; Centre for economic Analysis (CenEA), Szczecin, Zachodniopomorskie, Poland; Centre for Economic Analysis, CenEA, Szczecin, Zachodniopomorskie, Poland

## Abstract

We examine the responsiveness of later-life well-being measures to income using unique data, matching regional and individual level information from Germany and Poland. These two neighboring countries still strongly differ both in their welfare system designs, as seen in contrasting public social support as well as the quality of services, and in outcomes among older individuals. For example, the proportion of individuals aged 50+ who declare significant difficulties in making ends meet is only 3% in Germany, and as high as 16% in Poland. While up to 13% of individuals aged 65+ are classified as materially and socially deprived in Poland, the proportion in Germany is only 6%. Granted the importance of contextual factors in determining individual welfare, income is expected to matter less for the well-being of those enjoying higher quality local environments. Our preliminary findings confirm a much stronger relationship between income and well-being in Poland. The relationship is also found to be highly heterogeneous – for instance, it is substantially greater among those in poor health. Individual resources are thus shown to be more strongly related to well-being in conditions wherein individuals cannot rely on support through public services. Surprisingly, in both countries the effect of income on well-being does not significantly change when we control for the different local conditions expected to mediate the relationship. This suggests that the country-wide systemic context matters more for the examined relationship than specific regional environment.

